# The reliability of a new method of Intermetatarsal angle assessment in Hallux Abducto Valgus surgery

**DOI:** 10.1186/1757-1146-3-S1-O1

**Published:** 2010-12-20

**Authors:** Simon Adam

**Affiliations:** 1 Lambeth Community Health, London, UK

## 

This research forms the dissertation for an MSc in Podiatry.

The Intermetatarsal (IM) angle forms an integral component in the assessment of Hallux Abducto Valgus (HAV) surgery. Commonly used to assess the severity of such deformity and guide surgical selection, the degree of accuracy is debatable for both clinical and research purposes.

A common fault of previously described IM angle assessment techniques is that when the shaft of the metatarsal, or the metatarsal head its self has been altered through the surgery there is inaccuracy associated with the measurement of the post operative radiograph when these reference points are used.

A new method of assessment is proposed which can be applied for accurate assessment of both pre and post operative radiographs using the same method of examination. This new technique utilises the lateral most margins of the head of the metatarsal and most lateral margin of the base of the metatarsal. It is inherently applicable to all base, mid-shaft and distal osteotomy selections with or without medial eminence cheilectomy, a feature its predecessors have had difficulty in assessing accurately.

All patients whom underwent a surgical procedure to change the IM angle with in our surgical department were recruited into our study. There were no restrictions on the severity of deformity, osteotomy selection or preoperative examinations.

Pre and post operative radiographs were assessed blinded to the surgery three times by three different members of the surgical team. The inter- and intra-tester reliability of the new examination was compared against commonly used examination techniques.

It is anticipated that this new method of IM angle assessment will provide grounding for a uniform pre- and post-operative radiographic interpretation for a variety of surgical procedures. It is hoped this simple technique will be applicable to future research and clinical settings.

